# Relating the ultrasonic and aerosol filtration properties of filters

**DOI:** 10.1038/s41598-024-67809-w

**Published:** 2024-07-24

**Authors:** Tomás E. G. Alvarez-Arenas, Timothy A. Sipkens, Joel C. Corbin, Patricia Salso, Vicente Genovés

**Affiliations:** 1 Department of Ultrasonic and Sensors Technologies, Physical and Information Technologies Institute (ITEFI), Spanish National Research Council, Serrano 144, 28006 Madrid, Spain; 2 Metrology Research Centre, National Research Council Canada, 1200 Montreal Road, Ottawa, ON K1A 0R6 Canada

**Keywords:** Filtration efficiency, Air-coupled ultrasound, Air filters testing, Environmental sciences, Health care, Engineering, Materials science, Physics

## Abstract

Non-contact methods are useful to improve the quality control of particle filtration media. The purpose of this paper is to investigate the correlation between the filtration efficiency of a porous sheet and its ultrasonic properties obtained using a non-contact technique. An air-coupled ultrasonic technique is used to obtain rapid measurements without affecting the integrity of the material. High frequencies (from 0.1 to 2.5 MHz) are used to improve technique sensitivity, and transmitted waves are measured to probe the internal properties of the material. Measurements of transmission coefficient spectra (amplitude and phase) and the corresponding ultrasound velocity and attenuation coefficient at different frequencies are obtained for a set of filtration media with well-characterized properties. Results show that the ultrasonic properties of filtration media vary as a function of basis weight, and therefore filtration efficiency, for a given charge state. However, the effect of electrostatic charge on ultrasonic propagation is almost negligible, as expected. We conclude that ultrasonic transmission may provide a valuable tool for the continuous online monitoring of material quality during fabrication and as a method to tease apart mechanical and electrostatic contributions to particle filtration.

## Introduction

The filtration of aerosolized particles is critical to respiratory protection, within the context of both occupational and public health, and to building ventilation systems^1,2^. The demand for better, consistent quality filters requires methods to quickly ensure homogeneity and reproducibility of the materials that make up these products. This may become increasingly important as new filtration materials and new manufacturing methods are developed. Currently, measurements of particle filtration efficiency and pressure resistance require slow, typically offline, and typically destructive approaches to filter testing. Given that, in most cases, these materials can present some degree of intrinsic heterogeneity, testing large portions of the produced filters can become a key issue. This leaves an opportunity for complementing standard testing with non-destructive, online techniques.

Acoustic and ultrasonic techniques, encompassing both water and air-coupled approaches, have been used in the past to test different porous media, where ultrasonic and acoustic properties have been correlated with different material properties, including permeability, fluid filtration efficiency, tortuosity, porosity, and pore size. The relationship between permeability or fluid resistance and acoustic propagation in air saturated porous materials at audio frequencies is well understood and has been employed to characterize porous materials in different applications, especially for sound absorbers^3–9^. At the same time, fluid immersion ultrasonic techniques have also been used to study porous materials for fluid filtration—mainly filtration membranes—to determine membrane fouling during operation^10,11^ and particle retention capability^12^. Closer to the application here studied, ultrasonic waves and air-coupled techniques have also been used to study the correlation between permeability, flux resistivity, porosity and tortuosity with ultrasound propagation features for different porous and filtration materials^5,13–19^.

It has recently been demonstrated that, by using air-coupled ultrasonic transducers^20–22^ with high sensitivity, high center frequency (> 150 kHz), and large frequency band (fractional bandwidth at − 20 dB > 50%), it is possible to transmit ultrasonic signals through a variety of filtration layers used in the fabrication of face masks with sufficient signal-to-noise ratio to extract valuable information^23^. In addition, it was demonstrated that ultrasonic propagation takes place in the air gaps, such that ultrasonic parameters are sensitive to effective pore size, tortuosity, and porosity. In addition, it was shown that the loss in the transmission coefficient increases with the face mask grade, ranging from cloth face masks to respirators.

The purpose of this work is to apply the same air-coupled ultrasonic technique from a previous work^23^ to a collection of filtration materials with (1) controlled basis weight, (2) controlled charge applied to the material, and (3) whose particle filtration efficiency (PFE) and pressure resistance have also been measured using standard testing equipment^24^. This will provide a quantitative comparison between ultrasonic parameters and PFE. By controlling the electrostatic charge that have been applied to the materials, the effects thereof can be isolated and investigated. Results suggests that ultrasonic techniques may be useful as an online monitoring approach and may be capable of distinguishing between the mechanical and electrostatic filtration mechanisms.

## Results and discussion

### Material characterization

**Figure 1:**
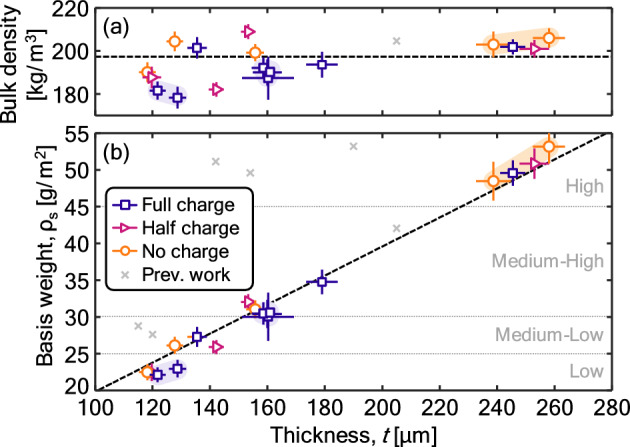
(**a**) Bulk density and (**b**) basis weight versus sample thickness. Dashed line corresponds to a linear regression of the basis weight data, forcing a y-intercept of zero, with the slope of that line corresponding to the horizontal rule in (**a**). Data from previous work^23,33^ include individual layers from a range of masks, including surgical masks and respirators. Whole masks had basis weights that exceeded the bounds here, e.g., surgical masks had basis weights of ρ_s_ ~ 70 g/cm^2^, consistent with other measurements^33^. Shading under the data connects repeat measurements.

Dimensional information, mass, particle filtration, and ultrasonic characteristics for the range of materials discussed in the Materials and methods section later in this manuscript are recorded in Table 1. The basis weight or surface density are used to categorize the materials. There is a close correlation between the categorical, target basis weight (used to group the samples in four categories) and the measured basis weight. Basis weight categories roughly correspond to 20–25 g/m^2^, 25–30 g/m^2^, 30–45 g/m^2^, and 45–55 g/m^2^ for the low, medium–low, medium–high, and high basis weight categories, respectively. A minor exception is material 6 (medium–low basis weight and full charge), which had a basis weight more consistent with the medium–high category. Figure 1 shows the basis weight as a function of material thickness. Basis weights versus thickness (Fig. 1b) are well-described by a linear trend line with respect to thickness that passes through the origin and has a slope of 198 ± 19 kg/m^3^. Such a trend corresponds to a constant bulk density, shown as a horizontal rule in Fig. 1a. There is some evidence of an increase in the bulk density with thickness, an indication that there may be a small increase in the apparent material density (and hence, porosity) with increasing thickness. However, these deviations are minor relative to the spread in the data.

**Table 1:** Properties of the samples, including thickness, density, and basis weight of the samples; particle filtration efficiency (PFE); and extracted ultrasonic parameters from the best fitting of the calculated spectra to the measurements.

Sample identification			Material characterization			Particle filtration testing			Derived ultrasonic properties				
ID	Charge^a^	Basis weight, category	Thickness [µm]	Density [kg/m^3^]	Basis weight, ρ_s_ [g/m^2^]	PFE^a^ [%]	Stdev/pentr. (–)	Pressure resistance, Δ*p* [Pa]^25^	Attenuation coefficient *a*_0_, [Np/mm]	Velocity, *v*_0_*,* [m/s]	n	m	Surface porosity, f, [%]
1	None	Low	118.2 ± 2.9	190.1 ± 4.6	22.5 ± 1.1	20.7	0.02	–	6.3	300	0.50	0	82.7
2	Half	Low	119.7 ± 3.2	187.7 ± 5.0	22.5 ± 1.2	65.1	0.05	–	6.1	285	0.55	0	83.0
3†	Full	Low	128.7 ± 3.0	178.3 ± 5.0	22.9 ± 1.2	83.4 (84)	0.07	42 ± 11	5.6	280	0.50	0	83.4
			121.8 ± 2.8	181.6 ± 4.1	22.1 ± 1.0				5.6	287	0.50	0	
4	None	Medium–Low	127.7 ± 2.8	204.5 ± 4.5	26.1 ± 1.2	25.2	0.02	–	6.6	274	0.55	0.02	81.4
5	Half	Medium–Low	142.2 ± 2.6	182.1 ± 3.3	25.9 ± 1.0	78.5	0.08	–	5.9	290	0.40	0.02	83.4
6†	Full	Medium–Low	160.0 ± 5.0	190.0 ± 6.0	30.4 ± 1.9	92.9 (94)	0.11	76 ± 23	7.9	245	0.50	0.06	82.7
			160.9 ± 3.0	190.0 ± 6.0	30.6 ± 1.9								
7	Full	Medium–Low	135.5 ± 3.3	201.4 ± 5.0	27.3 ± 1.3	89.3	0.12	–	7.1	280	0.55	0	81.7
8	None	Medium–High	155.7 ± 3.1	199.2 ± 3.9	31.0 ± 1.2	27.0	0.02	–	7.0	275	0.50	0.02	81.9
9	Half	Medium–High	153.3 ± 2.5	209.0 ± 3.4	32.0 ± 1.1	82.6	0.06	–	7.0	275	0.50	0.01	81.0
10	Full	Medium–High	179.0 ± 5.5	193.8 ± 6.0	34.7 ± 1.1	94.3	0.07	–	8.0	268	0.52	0.03	76.8
11†	Full	Medium–High	160.2 ± 9.0	187.4 ± 10.0	30.0 ± 3.3	90.6 (91)	0.11	61 ± 16	6.9	275	0.45	0	82.8
			158.5 ± 4.0	192.2 ± 4.8	30.5 ± 1.5				7.0	280	0.50	0.02	
12†	None	High	238.7 ± 6.0	203.2 ± 6.0	48.5 ± 2.7	41.7 (44)	0.04	94 ± 13	7.0	263	0.50	0.05	81.4
			258.1 ± 5.6	206.0 ± 4.5	53.2 ± 2.3				8.4	270	0.50	0.03	
13	Half	High	252.9 ± 5.1	201.0 ± 4.1	50.8 ± 2.1	86.4	0.07	–	7.0	270	0.55	0.04	81.7
14	Full	High	245.5 ± 4.3	201.9 ± 3.5	49.6 ± 1.7	94.4	0.06	–	7.2	268	0.50	0.05	81.6

^a^The no, half, and full charge terminology used in this manuscript corresponds directly to the no, medium, and high charge designations used in Sipkens et al.^24^.

^b^For PFE and pressure drop, some values are taken from a related interlaboratory comparison (ILC)^25^ that considered some of the same materials.

^†^Replicates were available for these cases, shown as separate values in the corresponding cells.

### Particle filtration efficiency and pressure resistance

**Figure 2:**
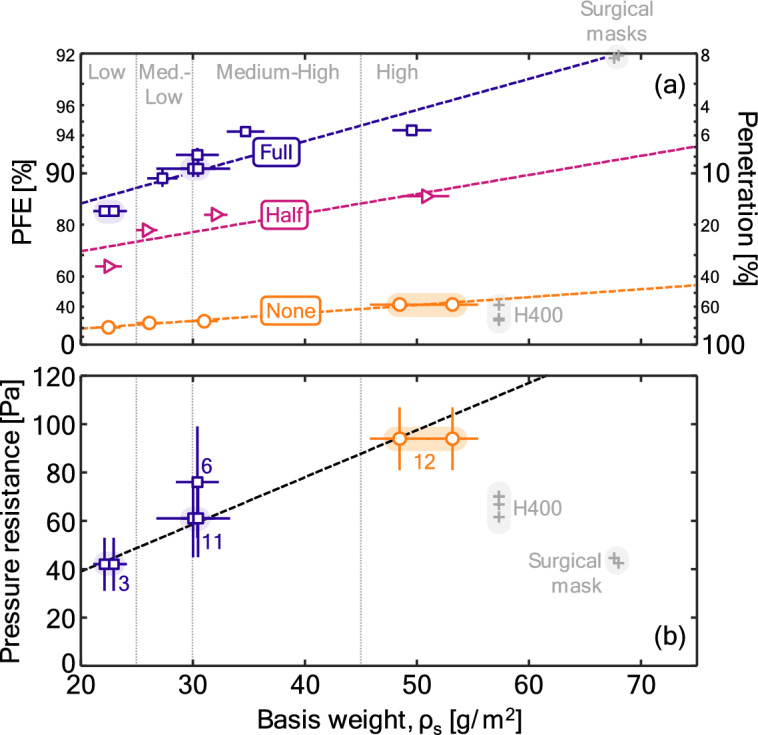
Variation in (**a**) PFE and (**b**) pressure resistance with the basis weight across the different charge states. Symbols indicate experimental data while dashed lines correspond to a linear fit for pressure resistance and realization of Eq. (3) at the different charge levels. H400 (a spunbond–metlblown–spunbond or SMS material) and surgical mask data are taken from Rogak et al.^33^, after integrating the measurements (noting distribution truncation errors and flow rate variations increase uncertainties). Pressure resistance values are corrected for flow rate from the original values assuming a linear relationship. Numbered labels in the pressure resistance panel corresponds to the IDs from Table 1 for the materials with replicates.

Figure 2 shows results of traditional measurements within the context of respiratory protection, namely PFE and pressure resistance, plotted as a function of the basis weight across the three different levels of charge. Note that the PFE is transformed prior to analysis, first by converting the PFE to penetration,

1 $$p=1-\eta ,$$

where η is the PFE is entered as a fraction (i.e., a PFE of 100% is entered as η = 1), and then taking the logarithm, to define

2 $$q=\text{ln}p=\text{ln}\left(1-\eta \right).$$

An expanded justification of this transformation is provided in Section "Particle filtration efficiency and pressure drop". Briefly, the choice both (i) is driven by the physics of filtration and (ii) has some precedent in the literature (via filter quality^28^).

PFE data is clearly clustered for each charge level. For the same range of basis weights, increasing the charge applied to the filter results in higher PFE, as expected (the presence of charge in the material enhances electrostatic particle capture). PFE data is fit using a second-order (with respect to charge) model allowing for first-order interactions between basis weight and charge state (see "Relating ultrasonic quantities to PFE"):

3 $$q={a}_{0}c+{a}_{1}{c}^{2}+{a}_{2}{\rho }_{\text{s}}+{a}_{3}c{\rho }_{\text{s}}+\text{ln}\left(1\right)$$

where *a*_0_, *a*_1_, *a*_2_, *a*_3_, and *a*_4_ are fitting coefficients; *c* is the charge, taking on values of 0, 0.5, and 1.0 for the no, half, and full charge cases, respectively; and *q* represents a transformation of the PFE, as per Eq. (2). This is a rather standard expression for multiple regression (regression within the context of multiple parameters, here basis weight and charge state). We note three things about the application here. First, the choice to add a second-order representation with respect to charge, while not expected to be physical, allows for a fully determined system (roughly, three equations, three unknowns with respect to charge) and thereby avoids introducing undue errors in the other coefficients in the absence of a physical model. However, we emphasize that, as a result, the coefficients *a*_0_ and *a*_1_ are not expected to have a physical interpretation. Second, the final term in this equation intuitively forces an uncharged filter with no basis weight (ρ_s_ = 0) to have a PFE of η = 0 (or, equivalently, *q* = ln 1), as this corresponds to when no filter is present. Enforcing this condition does little to change the fit. Finally, the choice to include an interaction term is necessary given that the slope with basis weights clearly changes with charge state.

Note that, for the no charge case (where *c* = 0), Eq. (3) reduces to

4 $$q={a}_{2}{\rho }_{\text{s}}+\text{ln}\left(1\right),$$

only incorporating one of the free parameters. The fit to the data is good, despite such a limited model. The adequacy of the fit suggests that the chosen transformation to the PFE is likely reasonable and, given that the no-charge case will be dominated by mechanical filtration (diffusion, impaction, and interception), suggests that the mechanical filtration scales in a predictable fashion with basis weight of the material.

Fits somewhat underperformed for the charged cases, where residuals have some limited structure. This discrepancy likely steams from differences in the size-dependent filtration when adding charging to a material^33^, which will introduce non-linearities when only considering an integrated PFE against the basis weights and will correlate better with only the mechanical filtration contributions.

Data for pressure drop demonstrates a roughly linear function of basis weight, consistent with Darcy’s Law. Uncertainties are significant relative to this trend, with sparse data limiting any substantial conclusions beyond a simple trend.

The materials considered here have lower basis weights than surgical masks^23^, which is expected given that surgical masks typically contain a similar meltblown layer but have additional minimally-filtering layers (typically a spunbond–meltblow–spunbond or SMS structure). The surgical masks had PFEs consistent with a charged material (noting uncertainties associated with differences in flow rate and size distribution truncation errors) and similar pressure resistances (ranges from 40 to 120 Pa^33–35^ after correction to the flow rate used here), though at the higher basis weights. The Halyard H400 wrap, another SMS material, also had similar pressure resistance^33^ but with a PFE more consistent with uncharged material, as expected.

### Ultrasonic results

**Figure 3:**
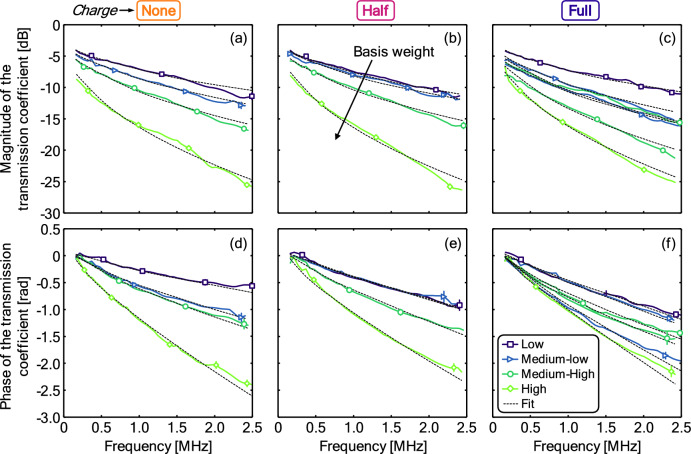
(**a**–**c**) Magnitude and (**d**–**f**) Phase of the transmission coefficient plotted against frequency. Lines correspond to fits to the data, with only select measurement errors shown to reduce clutter. Measurement errors generally increase with frequency. Solid lines correspond to measurements, while thinner dashed lines correspond to theoretical fits.

Ultrasonic material parameters are extracted by minimizing the sum- of- the squared differences between measured (see "Ultrasonic measurements") and calculated modelled (see "Ultrasonic model") spectra. Further details about the ultrasonic measurements including measured magnitudes and procedures can be found in the Materials and Methods section, in particular, in sections "Ultrasonic measurements" and "Ultrasonic model". Figure 3 shows the measured and calculated (a–c) magnitude and (d–f) phase of the transmission coefficient spectrum. Model fit is considered good considering the signal-to-noise ratio and some material variability.

#### Relationship between the ultrasonic quantities and basis weight

**Figure 4:**
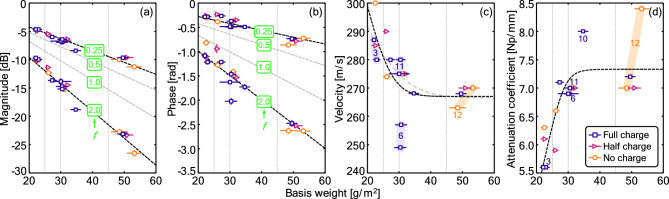
Variation in the (**a**) magnitude and the (**b**) phase of the transmission coefficient, (**c**) ultrasonic velocity, and (**d**) attenuation coefficient with the basis weight at three different charge levels. Dashed lines correspond to representative fits, with individual linear fits in (**a**,**b**) and an initial linear trend that saturates in (**c**,**d**). Numbers in (**a**) and (**b**) indicate the ultrasonic measurement frequency in MHz. Numbered labels in (**c**,**d**) correspond to the IDs from Table 1.

Here, we consider how the ultrasonic quantities change with basis weight. For a fixed material, increasing compactness (smaller or fewer pores) and thickness (corresponding to a larger basis weight) will act to change both the amplitude and phase of the transmitted waves. Figure 4a,b show that there is a clear correlation between both the magnitude and phase of the transmission coefficient and the basis weight. In both cases the slope of linear fits (constraining the intercept to pass through the origin, such that there is no change in the magnitude of phase for a filter of no thickness) to the data increase with increasing ultrasonic frequency. Reduction in the magnitude of the transmission coefficient is caused by a combination of: (1) the energy reflection at the sheet–air interfaces and (2) the attenuation of the ultrasonic wave in the sample, which depends on the sample thickness and the size of the pores (also quantifiable with the attenuation coefficient). The former contribution is primarily related to the porosity of the sheet, while the latter is determined by the pore size and the sheet thickness. The reduction in the phase of the transmission coefficient, by contrast, is determined by: (1) the thickness of the sheet and (2) the ultrasonic velocity. The latter factor is mainly determined by the pore tortuosity: the larger the tortuosity, the lower the velocity.

To eliminate the impact of sample thickness on the results, Fig. 4c,d add consideration of the ultrasonic velocity and the attenuation coefficient, both of which should be intrinsic properties of the material that are independent of the thickness of the sample. Note that these parameters are not direct measurements but are rather inferred from a fitting procedure (see "Ultrasonic model" and the results shown in Fig. 3). Figure 4c reveals an initial decrease in the ultrasonic velocity for low and medium–low basis weight materials. This suggests that the thinner samples exhibit some decreasing tortuosity with basis weight, while samples with higher basis weighted exhibit roughly constant tortuosity. Figure 4d similarly reveals an initial increase in the attenuation coefficient, which suggests that the pore size decreases with basis weight for low and medium–low basis weight materials. For samples with higher basis weights, there is some indication of a transition to a more constant attenuation coefficient, though considerable spread in the data makes such a conclusion uncertain. Material 6 (full-charge and medium–low basis weight) is an outlier in Fig. 4c. The thickness of this sample is also significantly larger than for the other medium–low samples, even if the density was in the same range. This may suggest some anomalies in this sample, consistent with the anomalous basis weight noted earlier in this work. Ultrasonic measurements suggest a smaller effective pore size and more tortuous structure, which is consistent with the higher value of pressure resistance (relative to the overall trend) in Fig. 2b.

Overall, trends are less significant for ultrasonic velocity and attenuation coefficient than for the magnitude and phase, which suggests that sample thickness is likely the leading driver of changes in the magnitude and phase of the transmission coefficient across much of the domain, which further justifies the choice of simple linear fitting for those scenarios.

In all cases, the influence of the charge on the ultrasonic measurements is very small, without any clear structure in the data in Fig. 4. Attenuation of the ultrasonic waves is produced by the viscous drag force that appears in the relative movement between the air in the pores and the solid frame under the action of the ultrasonic wave. As air molecules are largely free of charge, filter charging is expected to have a limited effect on this drag force, consistent with these observations. The contribution of the electrostatic charge to the variation of the ultrasonic velocity is likely even smaller, given that the contribution of electrostatic charge to the variation in the effective pore tortuosity will be very small. Given that sample thickness is also expected to be independent of charge, it is unsurprising that little structure appears in Fig. 4.

#### Relating ultrasonic quantities to PFE

**Figure 5:**
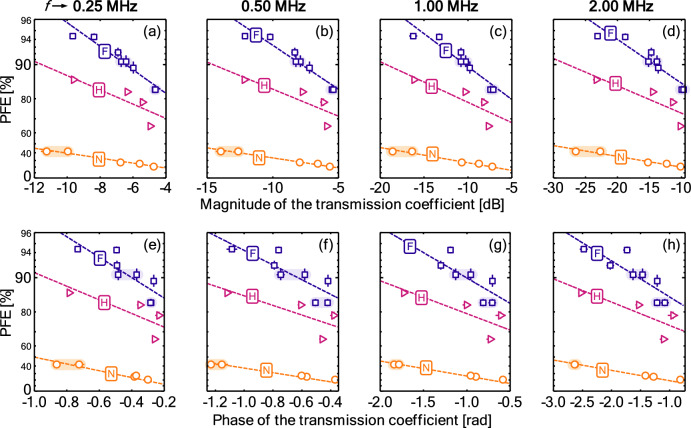
The PFE plotted against (**a**–**d**) magnitude and (**e**–**h**) phase of the transmission coefficient across the different charge levels. Symbols indicate experimental data, while dashed lines correspond to fits derived by fitting all of the data in each panel using a second-order interaction model (quadratic with respect to charge state allows for a full constrained regression in that dimension rather than representing the physics).

Figure 5 show the variation of PFE with the (a–d) magnitude and (e–f) phase of the ultrasonic transmission coefficient across the different acoustic frequencies and filter charge levels. In general, the data resembles the trends in the PFE with basis weight in Fig. 2. The observed trends are similar across the acoustic frequencies and the magnitude and phase of the transmission coefficient: the higher the PFE, the larger the loss in the magnitude and the larger the shift in the phase. As per section "Relationship Between the ultrasonic quantities and basis weight", reductions in the magnitude and phase are expected to be primarily driven by sheet thickness. Section "Particle filtration efficiency and pressure resistance" demonstrated that PFE is similarly driven by differences in basis weight. It is unsurprising, then, that there is a high degree of correlation between the PFE and the magnitude and phase. Slopes are smaller at the lower ultrasonic frequencies, indicating less sensitivity to the PFE.

The combination of Eq. (3) and the linear trends used in Fig. 4 would suggest fits of the form

5 $$q={b}_{0}c+{b}_{1}{c}^{2}+{b}_{2}x+{b}_{3}cx+\text{ln}\left(1\right)$$

where *x* is either the magnitude or the phase of the transmission coefficient, depending on the panel, and *b*_0_, *b*_1_, *b*_2_, and *b*_3_ are coefficients to be fit. Table 2 shows parameters from this fitting procedure.

**Table 2:** Parameters to predict PFE from the magnitude or phase of the transmission coefficient according to Eq. (5), as per Fig. 5.

	Frequency, *f* [MHz]	1st-order charge, *b*_0_	2nd-order charge, *b*_1_	Slope in *x*, *b*_2_	Charge-*x* interaction, *b*_3_
Magnitude	0.25	− 52.7	897	0.0504	5.67
	0.50	− 57.6	912	0.0408	4.14
	1.00	− 57.8	903	0.0310	3.08
	2.00	− 60.2	878	0.0219	2.02
Phase	0.25	− 78.3	1026	0.6889	41.79
	0.50	− 73.8	982	0.4674	30.62
	1.00	− 70.5	974	0.3049	21.78
	2.00	− 68.2	985	0.2103	15.68

Charge is normalized, having values of 0 (none), 0.5 (half), and 1.0 (full). The quantity x is either the magnitude or the phase of the transmission coefficient.

In terms of the level of charge, there is a clear relationship between the level of charge on the filter and both the slope and intercept of trends lines through the data. A higher charge on the filter results in a more rapid increase in the PFE for a similar reduction in the magnitude and phase. This follows from the fact that the PFE per unit thickness is higher for charge filter media.

**Figure 6:**
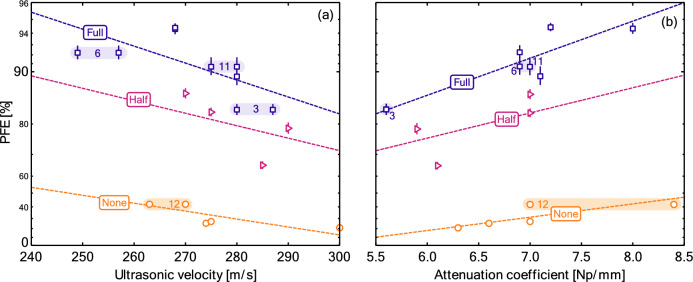
The PFE plotted against (**a**) the ultrasonic velocity and (**b**) attenuation coefficient. Symbols indicate experimental data, while dashed lines correspond to fits derived by fitting all of the data in each panel using a second-order interaction model and realized at each charge state. Numbered labels correspond to the IDs from Table 1 for the materials with replicates.

The combination of these observations would suggest that the ultrasonic quantities give a good measure of product thickness and the resultant mechanical filtration. In an attempt to remove some of the effect of the thickness, we also consider trends in with the ultrasonic velocity and attenuation coefficient in Fig. 6. The fitting procedure is analogous to those applied previously, e.g., in connection with Fig. 2. If there were no effects beyond thickness, theses plots should show slopes of zero, given that the ultrasonic velocity and attenuation constant should only contain noise from the fitting procedure. Rather, we see secondary effects where the ultrasonic velocity and attenuation coefficient are negatively and positively correlated, respectively, with PFE. For the velocity, the negative correlation suggests that higher PFE is associated with higher pore tortuosity in the material. Uncertainties remain significant here. Stratification with charge is still clearly evident, with the ultrasonic velocity and attenuation coefficient having a similar range across the different charge states. Interestingly, the slope of the curves does change. The precise reason for this remains unclear but may provide a route to further online assessment of materials. Nevertheless, this does suggest that the ultrasonic measurement can be used for qualitative validation of produced material.

#### Relating ultrasonic quantities to pressure resistance

**Figure 7:**
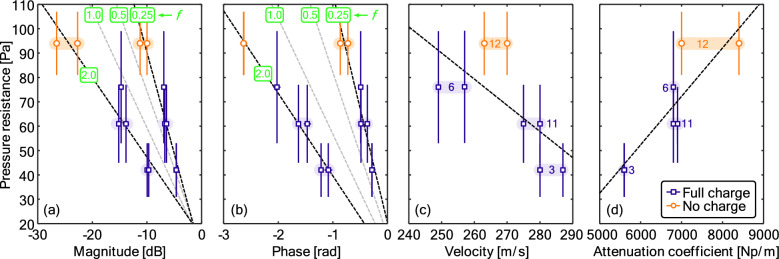
Variation in pressure resistance with (**a**) magnitude, (**b**) phase, (**c**) velocity, and (**d**) attenuation coefficient at different frequencies, (**f**) dashed lines indicate either constant or linear fits to the data. Numbered labels in (**c**,**d**) correspond to the IDs from Table 1 for the materials with replicates.

Figure 7a,b show the variation in the pressure resistance with the magnitude and the phase of the transmission coefficient, respectively, across a range of ultrasonic frequencies. In all cases, there is a clear correlation with pressure resistance, with higher frequencies inducing a stronger variation in the transmission. Figure 7c,d adds consideration of changes in the pressure drop with the ultrasonic velocity and attenuation coefficient, respectively. There are weak trends in both cases.

### Comparison between ultrasonic parameters

**Figure 8:**
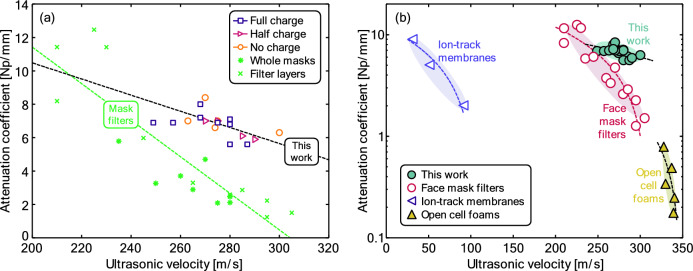
Ultrasonic attenuation coefficient at 1 MHz vs ultrasonic velocity and linear fitting. Black circles: Materials investigated in this work, Grey circles: face mask filters, taken from Ref.^23^, black triangles: open cell foams (taken from^36^) and black left-looking triangles: ion-track membranes (taken from^17,37,38^).

Figure 8 shows the relationship between the attenuation coefficient and the ultrasonic velocity. Figure 8a also shows measurements previously obtained for the filtration material of different face masks, spanning cloth masks, medical face masks, and respirators (KN95, FFP2)^23^. These materials have in common that the ultrasonic attenuation coefficient increases with *f *^*1/2*^, while the velocity is close to constant (see Table 2). The observed trends are similar between the two studies, but attenuation coefficients are larger for the materials studied in this case.

A broader comparison is shown in Fig. 8b. This includes other porous materials where velocity is not constant with the frequency and attenuation does not follow a *f *^*1/2*^ law. In particular, results for ion-track membranes and open cell foams are shown. In all cases, there is a linear relationship between ultrasonic velocity and attenuation coefficient. As expected, the larger the attenuation, the lower the velocity.

## Implications and future work

This work demonstrates a linear relationship between the ultrasonic transmission coefficient (both magnitude and phase) and the logarithm of the penetration (the converse of the particle filtration efficiency) of uncharged media as well as the pressure drop. The higher the frequency, the larger the variation in the transmission coefficient with both PFE and pressure drop. This indicates that it should be possible to use the ultrasonic transmission coefficient measurements as a fast estimator of mechanical (i.e., impaction, diffusion, and interception) contributions to PFE. The advantage of the air-coupled ultrasonic technique is that it is fast and non-destructive. Considering the basic experimental settings used in this work (a pulse repetition frequency or PRF of 1 kHz and averaging over 50 measurements), a single measurement is obtained in 50 ms. This sampling rate makes it possible to use this system for continuous online monitoring of material quality during fabrication. However, as will be discussed in "Ultrasonic measurements", it may be possible to further reduce the measurement time below 0.1 ms, when necessary, by optimizing the averaging procedure.

There is not a significant change in the ultrasonic properties of the materials in terms of variation in the electrostatic charge in the filter. The presence of electrostatic charge does impact the slope and the intercept of trends in the transmission coefficient, ultrasonic velocity, and attenuation coefficient with respect to PFE. As such, having a set of similar materials with homogeneous level of charge and graded PFE, it may be possible to infer the level of charge from the ultrasonic measurements or to confirm if the expected level of charge is achieved. Alternatively, the ultrasonic technique could be used to distinguish between the mechanical and electrostatic contributions to PFE. Again, this means that ultrasonic monitoring could provide a valuable method for the quality control of the mechanical properties of filtration media during fabrication.

The magnitude of the transmission coefficient presents a clear correlation with the reported pressure resistance; wherein results in the transmission coefficient magnitude and phase coincide with a higher pressure resistance.

Practical realization of this technique requires analysis of a larger set of samples to determine the broader effects of tortuosity on the PFE, preferably while still controlling for the basis weight and charge (e.g., using uncharged spunbond). Analysis of different types of materials—including fabrication method, microstructure, thickness, etc. —will contribute to further determine the versatility of the proposed method and step towards making the method quantitative. Alternatively, the technique could be applied directly for rapid, qualitative continuous monitoring of product quality. It may also be possible to use the technique to detect changes in filters during their use (e.g., monitoring of filters in HVAC systems). In both of these cases, the technique could benefit from the use of MEM (microelectromechanical) transducers, such as PMUT (piezoelectric micromechanized transducers) or CMUT (capacitive micromechanized transducers).

This work has not quantified the sensitive of the techniques to the effect of temperature and humidity on the samples. Future work could also investigate the effect of these external factors, on both the ultrasonic parameters and filtration properties, as there may be circumstances in which these properties may be more difficult to control (e.g., within an HVAC system).

## Materials and methods

### Materials and basic characterization

Materials consisted of meltblown polypropylene, a material often used in face mask construction, in the form of sheets (140 mm × 140 mm, pprox..) with controlled basis weight and levels of charge (by applying a varying degree of voltage to the material) and manufactured by Roswell Downhole Technologies (Alberta, Canada). A subset of the materials were also used by Sipkens et al.^24^ and in an interlaboratory comparison (ILC)^25^. Materials were grouped into four categories, depending on their target basis weight (Low, Medium–Low, Medium–High and High) and into three categories depending on the electrostatic charge (no, half, and full voltage, which correspond to the no, medium, and high charge levels, respectively, in Sipkens et al.^24^). About 20 sheets of each of the 14 samples were available and used for the ultrasonic measurements.

Ten measurements of thickness, mass, and surface area were taken in each type of sample, from which basis weight and density can be calculated. Thickness was measured using with a micrometre (Mitutoyo, Japan) and the mass was measured with an analytical balance (Precisa XT 220A).

### Particle filtration efficiency and pressure drop

Particle filtration efficiency (PFE) and pressure drop were measured using a TSI 8130A at the National Research Council Canada following the NIOSH TEB-APR-STP-0059 test procedure^26^, that is the procedure used to certify N95 respirators. This involves challenging the filtration media with a neutralized (charge-equilibrated) sodium chloride aerosol having a count median diameter of 75 ± 20 nm and a geometric standard deviation with respect to mobility diameter of σ_g_ ≤ 1.86. Typically, the aerosol is found to have a GSD close to this upper limit. The reported PFE corresponds to the initial filtration efficiency after a minute of testing. PFE is measured using a pair of photometers, which results in a PFE weighted to yield the integrated mass of particulate across a range of sizes captured by the filter, as discussed elsewhere^24,27^. Note that this quantity is integrated over the aforementioned size distribution, which can mask size-dependent effects. In all cases, a standard 85 lpm is used for measurements, which corresponds to a face velocity of ~ 10 cm/s for the filtering area in the instrument. Pressure resistance was measured in conjunction with an ILC^25^ using analogous settings to those above but spanning a larger range of instruments. PFE reported from that ILC is consistent with the measurements made in conjunction with this study.

As mentioned in "Particle filtration efficiency and pressure resistance" PFE is transformed prior to analysis: converting the PFE to penetration, Eq. (1), and then taking the logarithm, Eq. (2). The penetration is a more fundamental quantity, related directly to the reduction in number concentration due to the filter. Then, we note that the penetration for a given particle size is expected to follow an exponential function of thickness (and basis weight, given the aforementioned linearity between the thickness and basis weight), following from the assumption that each differential element of thickness in the filter reduces the number concentration by roughly the same fraction^28^. Similar statements hold for combining the different filtration mechanisms (e.g., mechanical versus electrostatic filtration, noting that these can have limitation associated with particle size). The logarithmic transform also corresponds to the quantity that, when combined with pressure resistance, is used to calculate the quality factor of a material^28^. Further, this quantity removes one of the constrains, here taking any value from – ∞ to 0 (when η = 0) instead of spanning from 0 to 1. Shifts in the penetration curve with respect to size, as happens when a subset of the filtration mechanisms is altered (e.g., electrostatic filtration is increased while holding the mechanical filtration constant), limit the accuracy of this approach. Nevertheless, the approach seems to be reasonable for the samples considered in this work.

### Ultrasonic measurements

**Figure 9:**
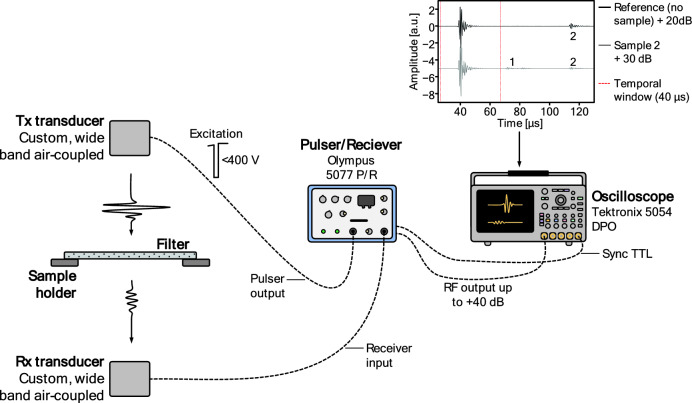
Schematic demonstrating the ultrasonic measurement setup. Dashed lines indicate signal cables. Tx denotes the transmitting transducer, while Rx denotes the receiving transducer, after the wave has transmitted through the filter. Inset panel shows an example signal received by the Rx for the 1.1 MHz pair of transducers. The black solid line (top) is the reference signal without a sample present; the grey solid line (bottom) denotes the signal when Sample 2 is between transducers at normal incidence; and the red dashed line denotes the 40 µs temporal window. Also visible are the first reverberation in the air-gap between Tx and Rx appears, labelled as “2” and occurring at 115 µs, as well as the first reverberation in the sample-Tx and sample-Rx gap, labelled as “1”. In this case, the Tx-Rx separation is 12 mm, the excitation amplitude is 200 V, the pulse repetition frequency (PRF) is 1 kHz, the gain in reception is + 20 dB for the reference signal and + 30 dB for the signal with the sample between Tx and Rx, the oscilloscope sampling frequency is 100 MS/s, the record length is 20 K, and the results are averaged over 50 repeats.

The transmission coefficient spectra (magnitude and phase) of airborne ultrasonic waves are measured in the frequency range 0.2–2.5 MHz, at normal incidence. A schematic representation of the experimental set-up is shown in Fig. 9. Four pairs of high sensitivity and wide band air-coupled ultrasonic transducers, designed and fabricated at the Spanish National Research Council (CSIC), are used with center frequencies of 0.25, 0.65, 1.1 and 2.0 MHz. They have overlapping frequency bands, and are used to cover this whole frequency range (0.20–0.35, 0.35–0.90, 0.70–1.30 and 1.20–2.50 MHz, respectively), with transducer aperture diameters of 25, 20, 15, and 15 mm, respectively. We refer the reader to Refs.^20–22^ for more general information on the hardware. In all cases the separation between transducers and sample is between 10 and 20 mm, where higher frequencies correspond to shorter transducer separations. All ultrasonic measurements were performed in the CSIC lab in Madrid, with temperature in the range of 20–22 °C and relative humidity between 45 and 55%.

The transmitter transducer (Tx) is excited using a semicycle of square wave tuned to the center frequency of the transducer. This signal was generated by an Olympus 5077 Pulser/Receiver (P/R). As noted above, the pulse repetition frequency (PRF) was set to 1 kHz. The signal in the receiver transducer (Rx) was amplified by the receiver stage of the Olympus 5077 P/R, and transferred to a digital scope (Tektronix DPO 7054) where it was digitized. A 40 µs rectangular gate around the transmitted pulse is used to filter out any reverberation (coming from the Tx/sample and Rx/sample cavities). The gated signal was padded with zeros at the end, up to a record length of 10,000 points, before a Fast Fourier Transform (FFT) was applied. The Olympus 5077 P/R also provided a signal to the oscilloscope input trigger to synchronize the transducer excitation with the signal acquisition in the scope. The oscilloscope and P/R setting used for each pair of transducers is detailed in Table 3. The inset in Fig. 9 shows an example signal at the receiver for the 1.1 MHz transducers with and without sample (Sample 2 in this case), alongside the temporal gate used to filter out reverberations that are visible in the right portion of the panel.

**Table 3:** Scope and pulser/receiver setting for both the pulse/receiver (P/R) and oscilloscope.

Transducers (MHz)	P/R 5077 setting				Oscilloscope setting		
	Voltage (V)	Frequency (MHz)	Gain (dB)		Sampling frequency (MS/s)	Record length	Averaging
			Reference	Measurement			
0.25	400	0.5	0	10	20	10k	50
0.65	200	1.0	0	10	100	20k	50
1.1	200	1.0	20	30	200	20k	50
2.0	200	2.0	20	30	500	25k	50

Signal-to-noise ratio (SNR) is improved by averaging, here over 50 signals. When combined with a pulse repetition frequency (PRF) of 1 kHz, each measurement takes 50/1000 s or 50 ms. Note that this time can be reduced by averaging over fewer signals or increasing the PRF. Given the center frequency and bandwidth of the transducers used here, the latter approach is rather simple. The SNR could alternatively be improved by increasing of the excitation amplitude, increasing the gain in receiver, introducing a pre-amplifier in the receiver, or changing the excitation waveform (to a chirp or a tone burst). In the limit, when no averaging is applied, the time to take a single measurement is determined by the separation Tx-Rx and the duration of the pulse. For the Tx-Rx distances and transducers used in this work, individual measurement durations (without averaging) span from 35 μs (high frequency) to 80 μs (low frequency).

The measured transmission coefficient is obtained by making two measurements. First, the FFT of the received pulse is obtained without a sample present between transmitter and receiver, following the aforementioned filtering of any reverberations. In this case, the gain in the receiver stage of the Olympus 5077 is set between 0 and 20 dB, as per Table 3. This measurement provides a calibration of the collective ultrasonic system, including the transmitter and receiver electronics, the response of the transducers, and the air gap between Tx and Rx (including the Tx/Rx distance and the temperature, pressure and humidity of the air). Second, a sample is measured at normal incidence, close to the midpoint of the transducers. The FFT of the received pulse is computed using the same rectangular gate used to filter reverberations as in the calibration process. In this case, the gain in the receiver stage of the Olympus 5077 is set between 10 and 30 dB, as per Table 3. Finally, the transmission coefficient spectrum is obtained from the division of these two spectra.

For each sample type, seven sheets were measured at the center point with the four pairs of transducers, allowing for an average and standard deviation for each case. The transmission coefficient was computed for each pair of transducers, then the measurements with the four pairs of transducers were merged. A continuous overlap of the measured transmission coefficient in the different frequency bands must be obtained. Finally, merged measurements for the whole frequency range were subsampled at 50 kHz, for display purposes.

### Ultrasonic model

According to Biot’s theory for the propagation of acoustic waves in fluid saturated porous media^29–31^, the transmission coefficient through the porous sheet at normal incidence can be described as:

6 $$T=\upphi {T}_{\text{f}}+\left(1-\upphi \right){T}_{\text{s}},$$

where *T*_s_ and *T*_f_ are the contributions of the fast wave and the slow wave propagation and ϕ is a factor that considers the mode conversion phenomena at the surface of the porous material^32^ that is related to the surface porosity, the pore aperture at the surface and the flux resistivity. In some cases, it can be assumed that the fast wave corresponds to the propagation in the solid phase, while the slow wave corresponds to the propagation in the fluid phase. As in Ref.^23^, one can then assume that all of the observed propagation takes place in the air in the fluid-filled space in the material and corresponds to the slow wave. In this case,

7 $$T=\upphi {T}_{\text{f}},$$

where *T*_f_ is taken for a flat layer, normal incidence, and unimodal propagation and is given by^23^

8 $${T}_{\text{f}}=\frac{2}{2\text{cos}kt+i\left(m+\frac{1}{m}\right)\text{sin}kt},$$

where *m* in Eq. 8 is the ratio of the impedances of the layer to the air; *t* the layer thickness; *k* the complex wave vector, *k*(ω) = ω/v(ω) + *i*·α(ω); α is the attenuation coefficient; *v* is the phase velocity, hereafter simply referred to as the *velocity*; and ω is the angular frequency of the ultrasonic wave.

Biot’s theory predicts that the slow wave velocity in the low frequency limit is given by

9 $$v={v}_{0}{\left(\frac{f}{{f}_{0}}\right)}^{0.5},$$

where *f* is the wave frequency. In the high frequency limit, the velocity is constant with the frequency and depends on the tortuosity, τ, as

10 $$v=\frac{{v}_{air}}{\sqrt{\tau }},$$

The tortuosity is defined as the ratio of actual flow path length to the straight distance between the ends of the flow path. Therefore, in the transition from the low to the high frequency limits, the variation in the velocity with the frequency can be represented by a general power law,

11 $$v \propto {f}^{m},$$

where 0 < *m* < 0.5, that is, *m* = 0 in the high frequency limit. The attenuation coefficient in the low frequency limit of Biot’s theory is given by

12 $$\alpha = \alpha_{0} \left( {\frac{f}{{f_{0} }}} \right)^{n} ,$$

where *n* = 0.5, according to the low frequency limit of Biot’s theory. These expressions are fit to the measured transmission, *T*, by minimizing the sum-of-square between the measurement and model using the Levenberg–Marquard algorithm and solved for the optimal *v*_0_, α_0_, ϕ, *m*, and *n.*

## Data Availability

The datasets generated during and/or analysed during the current study are available from the corresponding author on reasonable request.

## References

[1] Kelly, F. J. & Fussell, J. C. Improving indoor air quality, health and performance within environments where people live, travel, learn and work. *Atmos. Environ.* **200**, 90–109. 10.1016/j.atmosenv.2018.11.058 (2019). 10.1016/j.atmosenv.2018.11.058

[2] Morawska, L. *et al.* A paradigm shift to combat indor resiratory infection. *Science* **372**, 689–691. 10.1126/science.abg2025 (2021). 33986171 10.1126/science.abg2025

[3] Delany, M. E. & Bazley, E. N. Acoustical properties of fibrous absorbent materials. *Appl. Acoust.* **3**, 105–116 (1970). 10.1016/0003-682X(70)90031-9

[4] Allard, J. F. & Atalla, N. Propagation of Sound in Porous Media. *Wiley*10.1002/9780470747339 (2009). 10.1002/9780470747339

[5] Álvarez-Arenas, T. E. G. Generation of the slow wave to characterize air filled porous fabrics. *J. Appl. Phys.* **78**(4), 2843–2845 (1995). 10.1063/1.360714

[6] Fellah, Z. E. A *et al.* Measuring flow resistivity of porous materials at low frequencies range via acoustic transmitted waves. *J. Acoust. Soc. Am.*, **119**(4), 10.1121/1.2179749ï. (2006). 10.1121/1.2179749 16642801

[7] Fellah, Z. E. A. Measuring permeability of porous materials at low frequency range via acoustic transmitted waves. *Rev. Sci. Instrum.* **78**(11), 114902–114902. 10.1063/1.2804127 (2007). 18052497 10.1063/1.2804127

[8] Yang, T. *et al.* Investigation on acoustic behavior and air permeability of struto nonwovens. *Fibers Polym.* **17**(12), 2078–2084. 10.1007/s12221-016-6967-9 (2016). 10.1007/s12221-016-6967-9

[9] Horoshenkov, K. V. A review of acoustical methods for porous material characterisation. *Int. J. Acoust. Vib.* **22**(1), 92–103. 10.20855/ijav.2017.22.1455 (2017). 10.20855/ijav.2017.22.1455

[10] Li, J. X., Sanderson, R. D. & Chai, G. Y. A focused ultrasonic sensor for in situ detection of protein fouling on tubular ultrafiltration membranes. *Sens Actuators B Chem.* **114**(1), 182–191. 10.1016/j.snb.2005.04.041 (2006). 10.1016/j.snb.2005.04.041

[11] Mairal, A. P., Greenberg, A. R., Krantz, W. B. & Bond, L. J. Real-time measurement of inorganic fouling of RO desalination membranes using ultrasonic time-domain reflectometry. *J. Membr. Sci.* **159**, 185–196. 10.1016/S0376-7388(99)00058-7 (1999). 10.1016/S0376-7388(99)00058-7

[12] Tran, D. H. *et al.* Ultrasonic measurements of particle retention by a porous medium. *Ultrasonics* **52**, 62–68. 10.1016/j.ultras.2011.06.011 (2012). 21788058 10.1016/j.ultras.2011.06.011

[13] Álvarez-Arenas, T. E. G. A nondestructive integrity test for membrane filters based on air-coupled ultrasonic spectroscopy. *IEEE Trans. Ultrason. Ferroelectr. Freq. Control* **50**(6), 676–685 (2003). 12839180 10.1109/TUFFC.2003.1209555

[14] Álvarez-Arenas, T. E. G. Air-coupled ultrasonic spectroscopy for the study of membrane filters. *J. Membr. Sci.* **213**, 195–207. 10.1016/S0376-7388(02)00527-6 (2003). 10.1016/S0376-7388(02)00527-6

[15] Fellah, Z. E. A. *et al.* Measuring the porosity and the tortuosity of porous materials via reflected waves at oblique incidence. *J. Acoust. Soc. Am.* **113**(5), 2424–2433. 10.1121/1.1567275 (2003). 12765361 10.1121/1.1567275

[16] Fellah, Z. E. A. *et al.* Determination of transport parameters in air-saturated porous materials via reflected ultrasonic waves. *J. Acoust. Soc. Am.* **114**(5), 2561–2569. 10.1121/1.1621393 (2003). 14649992 10.1121/1.1621393

[17] Álvarez-Arenas, T. E. G., Apel, P. Y. & Orelovitch, O. Ultrasound attenuation in cylindrical micro-pores: Nondestructive porometry of ion-track membranes. *IEEE Trans. Ultrason. Ferroelectr. Freq. Control* **55**(11), 2442–2449. 10.1109/TUFFC.951 (2008). 19049923 10.1109/TUFFC.951

[18] Álvarez-Arenas, T. E. G. & González Gómez, I. High-frequency tortuosity relaxation in open-cell foams. *IEEE Trans. Ultrason. Ferroelectr. Freq. Control* **56**(4), 772–778. 10.1109/TUFFC.2009.1099 (2009). 10.1109/TUFFC.2009.1099 19406705

[19] Fellah, Z. E. A. *et al.* Ultrasound measuring of porosity in porous materials, porosity—process, technologies and applications. *InTech*10.5772/intechopen.72696 (2018). 10.5772/intechopen.72696

[20] Álvarez-Arenas, T. E. G. Acoustic Impedance Matching of Piezoelectric Transducers to the Air. *IEEE Trans. Ultrason. Ferroelectr. Freq. Control* **51**(5), 624–633 (2004). 15217239 10.1109/TUFFC.2004.1320834

[21] Kelly, S. P., Hayward, G. & Álvarez-Arenas, T. E. G. Characterization and assessment of an integrated matching layer for air-coupled ultrasonic applications. *IEEE Trans. Ultrason. Ferroelectr. Freq. Control* **51**(10), 1314–1323. 10.1109/TUFFC.2004.1350960 (2004). 15553516 10.1109/TUFFC.2004.1350960

[22] Álvarez‐Arenas, T. E. G. Air‐coupled Ultrasonic Transducers. *Ultrasound in Food Processing*, pp. 175–228 (Wiley, 2017). 10.1002/9781118964156.ch7.

[23] Álvarez-Arenas, T. E. G., Fariñas, M. D. & Ginel, A. Fast and non-destructive ultrasonic test for face masks. *Ultrasonics*, **117**. 10.1016/j.ultras.2021.106556 (2021). 10.1016/j.ultras.2021.106556 34467874

[24] Sipkens, T. A. *et al.* Comparison of measurement systems for assessing number- and mass-based particle filtration efficiency. *J. Occup. Environ. Hyg.* **19**, 629–645. 10.1080/15459624.2022.2114596 (2022). 35994755 10.1080/15459624.2022.2114596

[25] Sipkens, T. A., *et al*. Report on the 2nd inter-laboratory comparison of filtration media testing equipment.

[26] Determination of particulate filter efficiency level for N95 series filters against solid particulates for non-powered, air-purifying respirators standard testing procedure (STP), https://www.cdc.gov/niosh/npptl/stps/pdfs/TEB-APR-STP-0059-508.pdf (2019).

[27] Biermann, A. H. & Bergman, W. Filter penetration measurements using a condensation nuclei counter and an aerosol photometer. *J. Aerosol. Sci.* **29**, 471–483 (1988). 10.1016/0021-8502(88)90022-5

[28] Hinds, W. C. & Zhu, Y. *Aerosol technology: properties, behavior, and measurement of airborne particles* (John Wiley & Sons, 2022).

[29] Biot, M. A. Theory of propagation of elastic waves in a fluid-saturated porous solid. I. Low-frequency range. *J. Acoust. Soc. Am.* **28**, 168–178 (1956). 10.1121/1.1908239

[30] Biot, M. A. Theory of propagation of elastic waves in a fluid-saturated porous solid. II. Higher frequency range. *J. Acoust. Soc. Am.* **28**, 179–191. 10.1121/1.1908241 (1956). 10.1121/1.1908241

[31] Biot, M. A. Generalized theory of acoustic propagation in porous dissipative media. *J. Acoust. Soc. Am.* **34**, 1254–1264 (1962). 10.1121/1.1918315

[32] Alvarez-Arenas, T. E. G., Apel, P. Y., Orelovitch, O. L. & Mitrofanov, A. V. Ultrasound propagation in the micropores of track membranes. *Appl. Phys. Lett.* **87**, 111911 (2005). 10.1063/1.2045542

[33] Rogak, S. N. *et al.* Properties of materials considered for improvised masks. *Aerosol. Sci. Technol.* **55**, 398–413. 10.1080/02786826.2020.1855321 (2021). 10.1080/02786826.2020.1855321

[34] Zangmeister, C. D., Radney, J. G., Vicenzi, E. P. & Weaver, J. L. Filtration efficiencies of nanoscale aerosol by cloth mask materials used to slow the spread of SARS-CoV-2. *ACS Nano* **14**, 9188–9200. 10.1021/acsnano.0c05025 (2020). 32584542 10.1021/acsnano.0c05025

[35] Drewnick, F. *et al.* Aerosol filtration efficiency of household materials for homemade face masks: Influence of material properties, particle size, particle electrical charge, face velocity, and leaks. *Aerosol Sci. Technol.* **55**, 63–79. 10.1080/02786826.2020.1817846 (2021). 10.1080/02786826.2020.1817846

[36] Alvarez-Aenas, T. E. G. & Diez, L. Novel impedance matching materials and strategies for air-coupled piezoelectric transducers, *Sens. 2013 IEEE*, 1–4. 10.1109/ICSENS.2013.6688505 (2013).

[37] Álvarez-Arenas, T. E. G., Apel, P. Y. & Orelovitch, O. Ultrasound attenuation in cylindrical micro-pores: Nondestructive porometry of ion-track membranes. *IEEE Trans. Ultrason. Ferroelectr. Freq. Control* **55**, 2442–2449. 10.1109/TUFFC.951 (2008). 19049923 10.1109/TUFFC.951

[38] Álvarez-Arenas, T. E. G., Apel, P. Y. & Orelovich, O. L. Characterization of ion-track membranes by non-contact ultrasonic magnitude and phase spectroscopy. *J. Membr. Sci.* **301**, 210–220. 10.1016/j.memsci.2007.06.024 (2007). 10.1016/j.memsci.2007.06.024

